# Placental dysfunction is associated with altered microRNA expression in pregnant women with low folate status

**DOI:** 10.1002/mnfr.201600646

**Published:** 2017-03-21

**Authors:** Bernadette C. Baker, Fiona L. Mackie, Samantha C. Lean, Susan L. Greenwood, Alexander E. P. Heazell, Karen Forbes, Rebecca L. Jones

**Affiliations:** ^1^ Maternal and Fetal Health Research Centre University of Manchester Manchester UK; ^2^ Centre of Women's and Newborn's Health & Institute of Metabolism and Systems Research University of Birmingham Birmingham UK; ^3^ Division of Reproduction and Early Development Leeds Institute of Cardiovascular and Metabolic Medicine University of Leeds Leeds UK

**Keywords:** fetal growth, folate deficiency, gene expression, microRNA, placental dysfunction

## Abstract

**Scope:**

Low maternal folate status during pregnancy increases the risk of delivering small for gestational age (SGA) infants, but the mechanistic link between maternal folate status, SGA, and placental dysfunction is unknown. microRNAs (miRNAs) are altered in pregnancy pathologies and by folate in other systems. We hypothesized that low maternal folate status causes placental dysfunction, mediated by altered miRNA expression.

**Methods and results:**

A prospective observational study recruited pregnant adolescents and assessed third trimester folate status and placental function. miRNA array, QPCR, and bioinformatics identified placental miRNAs and target genes. Low maternal folate status is associated with higher incidence of SGA infants (28% versus 13%, *p* < 0.05) and placental dysfunction, including elevated trophoblast proliferation and apoptosis (*p* < 0.001), reduced amino acid transport (*p* < 0.01), and altered placental hormones (pregnancy‐associated plasma protein A, progesterone, and human placental lactogen). miR‐222‐3p, miR‐141‐3p, and miR‐34b‐5p were upregulated by low folate status (*p* < 0.05). Bioinformatics predicted a gene network regulating cell turnover. Quantitative PCR demonstrated that key genes in this network (zinc finger E‐box binding homeobox 2, v‐myc myelocytomatosis viral oncogene homolog (avian), and cyclin‐dependent kinase 6) were reduced (*p* < 0.05) in placentas with low maternal folate status.

**Conclusion:**

This study supports that placental dysfunction contributes to impaired fetal growth in women with low folate status and suggests altered placental expression of folate‐sensitive miRNAs and target genes as a mechanistic link.

**Abbreviations**
CDK6: cyclin‐dependent kinase 6
FGR: fetal growth restriction
hCG: human chorionic gonadotropin
hPL: human placental lactogen
IBC: individualized birthweight centile
miRNA: microRNA
MYC: v‐myc myelocytomatosis viral oncogene homolog (avian)
PAPP‐A: pregnancy‐associated plasma protein A
RBC: red blood cell
SGA: small for gestational age
TEENS: Teenage Nutrition Study
TP53: tumor protein p53
VEGFA: vascular endothelial growth factor A
ZEB: zinc finger E‐box binding homeobox

## 1 Introduction

Poor maternal nutritional status increases the risk of adverse pregnancy outcomes, particularly fetal growth restriction (FGR) or small for gestational age (SGA) infants 1. Critically, this links maternal nutritional state with increased risk of stillbirth and neonatal death, as well as intrapartum and postnatal complications, and neurodevelopmental problems in childhood 2, 3. In addition, there is a strong association between SGA birth and higher risk of diseases in adulthood, including cardiovascular disease and metabolic syndrome 4, 5.

The mechanisms underlying increased susceptibility to FGR/SGA are unclear; while calorific and macronutrient insufficiencies may have direct consequences on fetal growth, inadequate levels of micronutrients are likely to have more complex indirect effects, either on the fetus or placenta 6. Folate is a critical nutrient for one carbon metabolism that produces nucleotide bases for DNA synthesis and repair, and the universal methyl donor S‐adenosylmethionine for methylation processes 7. Folate deficiency results in impaired cell survival and division, genomic instability, and dysregulated DNA/protein methylation. Therefore, adequate folate supply is critical during pregnancy, not only for preventing neural tube defects, but for normal fetal growth and development. Indeed, inadequate maternal folate concentrations are related to the birth of SGA infants 8, 9, 10.

Pregnant adolescents are a population susceptible to low folate status, due at least in part to inadequate dietary folate consumption; this in turn is strongly related to risk of SGA birth in this population 8. Despite this association, the mechanisms responsible are unclear. Placental dysfunction is an established underlying cause of SGA 11, thus we hypothesized that inadequate third trimester maternal folate status impacts fetal growth by adversely affecting placental function during the period of maximal fetal growth.

To test this hypothesis, we collected maternal serum and placental samples from well‐characterized adolescent pregnancies and analyzed placental function according to third trimester maternal folate status. We investigated placental expression of known and novel folate‐sensitive microRNAs (miRNAs) as a potential posttranslational mechanism linking low maternal folate status and placental dysfunction. Differential expression of placental miRNAs has been reported in FGR and in vitro studies have shown various miRNAs affect trophoblast proliferation, invasion, and survival 12. There are no studies in pregnancy linking folate status and miRNA expression, but in vivo and in vitro studies in other systems have demonstrated that folate deficiency alters miRNA expression 13, 14. The current study identifies a key involvement of the placenta in mediating the adverse effects of low maternal folate status on the developing fetus, and highlights dysregulation of placental miRNAs as a potential underpinning mechanism.

## 2 Materials and methods

### 2.1 Teenage Nutrition Study

Pregnant adolescents (16–18 years, *n* = 77) were recruited to Teenage Nutrition Study (TEENS) between 28 and 40 weeks gestation from St Mary's Hospital or local community antenatal clinics, Manchester. Exclusion criteria included multiple pregnancy, known fetal anomalies, or obstetric/medical complications, which may affect nutritional status. At recruitment, maternal venous blood was taken for endocrine analyses and to assess folate status. Demographic data, obstetric history, and lifestyle information including self‐reported folic acid/nutritional supplementation were recorded. Obstetric outcome data were collected and the individualized birthweight centile (IBC) was calculated using GRO Weight Centile Calculator version 7.4.2 (Gestation Network 2009). SGA was defined as IBC < 10th centile 15. The TEENS study was granted ethical approval by the North West REC (08/H1010/55). Written informed consent was obtained from all participants. Placentas from participants in the TEENS study were collected at delivery and used for all described analyses of placental function. Data and maternal serum samples from a further cohort of pregnant adolescents (*n* = 80, selected based on third trimester red blood cell (RBC) folate concentrations), previously recruited to the About Teenage Eating study (03/CM/32) 8, were included for obstetric outcome and endocrine analyses only. Placental samples were not obtained from this cohort. The same inclusion and exclusion criteria applied.

### 2.2 Analysis of maternal folate status

Maternal folate status was assessed as RBC folate (reflective of long‐term tissue folate content 16) and serum folate by the Clinical Biochemistry Department at Manchester Royal Infirmary. Folate deficiency is defined as serum folate <10 nmol/L or RBC folate <340 nmol/L based on elevated plasma homocysteine as a metabolic indicator of deficiency 17. Whether these thresholds apply to pregnant women is disputed, as folate demands are elevated in pregnancy and the association between low folate and SGA extends beyond this threshold 8. On the basis of our previous findings 8, and that folate concentrations below 453 nmol/L (200 μg/L) are clinically treated in our obstetric population, we defined low folate status as RBC <453 nmol/L.

### 2.3 Placental collection and sampling

Placentas were collected from a subgroup of TEENS participants (*n* = 46) within 30 min of delivery, and trimmed placental weight recorded. Villous tissue samples (2 cm^3^) were excised using a systematic uniform random sampling system 18 from four placental locations to minimize regional variation in gene expression 19. Samples were pooled prior to further dissection and randomly subdivided for histological, molecular, and nutrient transport analyses. A nested case–control study was utilized for placental analyses, with low and adequate folate status cohorts selected to match most closely on demographic and biophysical characteristics. Sample sizes were dictated by power calculations based on previous studies 20.

### 2.4 Immunohistochemistry

Immunohistochemistry was performed on 5 μm sections of formalin‐fixed wax‐embedded villous tissue from three regions/placenta using colorimetric detection as described 21. Mouse monoclonal primary antibodies used to detect proliferation and apoptosis were anti‐Ki67 (0.16 μg/mL, Dako, Ely, UK) and anti‐M30 (1 μg/mL, Roche, Hertfordshire, UK), respectively, with nonimmune mouse IgG as a negative control. Ten random images per section were captured using an Olympus BX41 microscope (Olympus, UK), QICAM Fast 1394 camera (QImaging, Canada), and Image Pro‐Plus version 7.0 (Bethesda, Media Cybernetics). Proliferative and apoptotic indices were calculated by manual counting of Ki67^+^ or M30^+^ trophoblasts divided by total nuclear count 21.

### 2.5 System A activity

Sodium‐dependent uptake of ^14^C‐methylaminoisobutyric acid (PerkinElmer, Buckinghamshire, UK) was measured to assess amino acid transporter system A activity in placental fragments as described 20. The rate of uptake over 10–30 min was calculated and normalized for fragment protein content.

### 2.6 Placental endocrine function

ELISAs for placental hormones (human chorionic gonadotropin (hCG), progesterone, human placental lactogen (hPL), and pregnancy‐associated plasma protein A (PAPP‐A) were performed on third trimester maternal serum (low folate: *n* = 57, adequate folate: *n* = 100), according to manufacturer's instructions (DRG Diagnostics, Germany), using a Versamax plate reader (Molecular Devices, CA, USA) at 450 nm using SoftMax Pro (Molecular Devices). Inter‐ and intraassay variabilities were 4.3–9.9% and 2.6–5.5%, respectively.

### 2.7 Real‐time PCR

Real‐time PCR was performed to quantify expression of genes encoding system A transporters, placental hormones, or predicted miRNA targets. Placental RNA (200 ng, RNAeasy kit; Qiagen) was reverse transcribed (AffinityScript multiple temperature cDNA synthesis kit, Agilent, Berkshire, UK) and QPCR performed using Brilliant SYBRIII Green QPCR Master Mix (Agilent) with 5‐carboxy‐x‐rhodamine as the reference dye in a Stratagene MX3000P real time PCR machine. Specific primers used are presented in Supporting Information Table 1. Data were normalized for expression of housekeeping gene TATA box binding protein as 2^−ΔCT^.

Candidate miRNAs were amplified by QPCR in placental RNA samples (Supporting Information Table 2, n = 11/group), extracted using *mir*Vana miRNA isolation kit (Ambion), and selected based on known regulation by folate (miR222, miR22, miR122, and miR302a). Reverse transcription of RNA (25 ng) was performed using miRCURY™ LNA Universal RT microRNA PCR system (Exiqon, Vedbaek, Denmark), with a UniSp6 RNA spike‐in template included as an internal QC to monitor the reaction efficiency QPCR was carried out using ExiLENT SYBR Green master mix (Exiqon) with LNA‐specific primer sets (Exiqon; Supporting Information Table 3) and reference dye 5‐carboxy‐x‐rhodamine. Data were normalized to 5S rRNA or U6 snRNA as housekeeping genes as 2^−ΔCT.^

### 2.8 miRNA array profiling

miRNA profiling on a subset of samples from TEENS with adequate and low folate status (Supporting Information Table 4, *n* = 7/group) was performed by Exiqon using the miRCURY LNA™ microRNA Array 7th Gen (Exiqon), containing capture probes targeting all human miRNAs registered in miRBASE 18.0 (Vedbaek, Denmark). Benjamini and Hochberg multiple testing correction was applied, using the software R/bioconductor, to calculate the adjusted *p*‐values where a value <0.05 was considered significant 22. QPCR validation studies on selected candidate miRNAs were performed as above on the same sample set used in section 2.7 for targeted analyses of folate‐sensitive miRNAs.

### 2.9 Bioinformatic analysis of miRNA array targets

Network generation was performed on identified folate‐regulated miRNAs using Ingenuity Knowledge Base (Ingenuity® Pathway Analysis, Qiagen, Redwood City, www.qiagen.com/ingenuity) to identify potential gene targets of the miRNAs and explore their connectivity, filtering to only include experimentally validated data. Experimentally validated gene targets of selected miRNAs were retrieved from high‐quality manually curated literature databases—miRTarBase Release 4.5 23 and TARBASE version 7.0 24.

### 2.10 Statistics

Statistical analyses were performed using GraphPad Prism (version 6.0). Demographic and obstetric outcome data were analyzed using Mann–Whitney *U* test, Spearman's correlation or Fisher's exact test. Data from placental and serum analyses were analyzed using Mann–Whitney *U* test, Spearman's correlation, or linear regression. Results were considered significant if *p* < 0.05.

## 3 Results

### 3.1 Demographic data

The demographic characteristics of the total study participants (TEENS and About Teenage Eating, *n* = 157) are present in Table 1. As per experimental design, both serum and RBC folate concentrations were different between groups (*p* < 0.0001) with no other differences apparent. The participants included in placental analyses (only from the TEENS study) were matched for demographic characteristics and did not differ from those of the total study group, with the exception of maternal BMI at time of booking to antenatal care, which was lower in women with low folate status (*p* < 0.05; Supporting Information Table 5).

**Table 1:** Demographic data of overall study participants

Category	Adequate folate status (*n* = 100)	Low folate status (*n* = 57)	*p*
Age (years)	18 (15–18.8)	17.7 (15–18.9)	NS
Gynecological age (years)	4.9 (1.0–9.6)	5.0 (2.0–9.1)	NS
Ethnicity *n* (%)			
Caucasian	69 (69%)	33 (57.9%)	NS
Other	31 (31%)	24 (42.1%)	N
Primiparous *n* (%)	94 (94%)	56 (98.2%)	NS
Smoking status *n (%)*			
Smoker	31 (31%)	24 (42.1%)	NS
Non‐smoker	69 (69%)	33 (58.9%)	NS
BMI (kg/m^2^)	22.5 (14.8–42.3)	22.3 (15.4–40.8)	NS
Gestation at delivery (weeks)	40.1 (35.9–42.0)	40.3 (36.0–43.1)	NS
Birthweight (g)	3300 (2020–4460)	3200 (2000–4160)	NS
Individualized birthweight centile (IBC)	40 (0–99)	28 (0–98)	0.05
Male infant *n* (%)	45 (45%)	27 (47.5%)	NS
Serum folate (nmol/L)	14.7 (2.7–51.0)	7.7 (3.4–17.0)	<0.0001
RBC folate (nmol/L)	948.3 (455.5–1602.1)	385.2 (219.8–449.0)	<0.0001

Data are median (range).Mann–Whitney or Fisher's exact test.

RBC, red blood cell.

### 3.2 Low folate status and pregnancy outcome

Adolescents with low folate status delivered infants with a lower IBC (*p* < 0.05) and a higher proportion of SGA infants than those with adequate folate status (32% versus 13%, *p* < 0.05) (Table 2). RBC folate concentration and IBC positively correlated (*r* = 0.2164, *p* < 0.01). No significant differences were detected in unadjusted birthweight, low birthweight <2500 g, large for gestational age infants, or gestation at delivery. Placental weight was unaffected by maternal folate status (not shown).

**Table 2:** Relationship between third trimester maternal RBC folate status and pregnancy outcome

	Adequate (*n* = 100)	Low (*n* = 57)	*p*	Correlation	*p*
Birthweight (g)	3300	3200	NS	0.04051	NS
	(2020–4460)	(2000–4160)			
IBC	39.9	27.8*	0.05	0.2164**	<0.01
	(0–99)	(0–98)			
Gestation at delivery	40.1	40.3	NS	–0.1083	NS
	(35.9–42.0)	(36.0–43.1)			
SGA *n* (%)	13	18**	<0.01		
	(13.1%)	(32.1%)			
LGA n (%)	7	4	NS		
	(7.0%)	(7.1%)			
LBW n (%)	5	8	0.068		
	(5.1%)	(14.3%)			

Median (range) value stated. Groups compared using Fisher's exact or Mann–Whitney test; relationship between continuous variables analyzed with Spearman's correlation. *p < 0.05, **p < 0.01.

IBC, individualized birthweight centile; SGA, small for gestational age; LGA, large for gestational age; LBW, low birthweight.

### 3.3 Impact of maternal folate status on placental cell turnover

**Figure 1:**
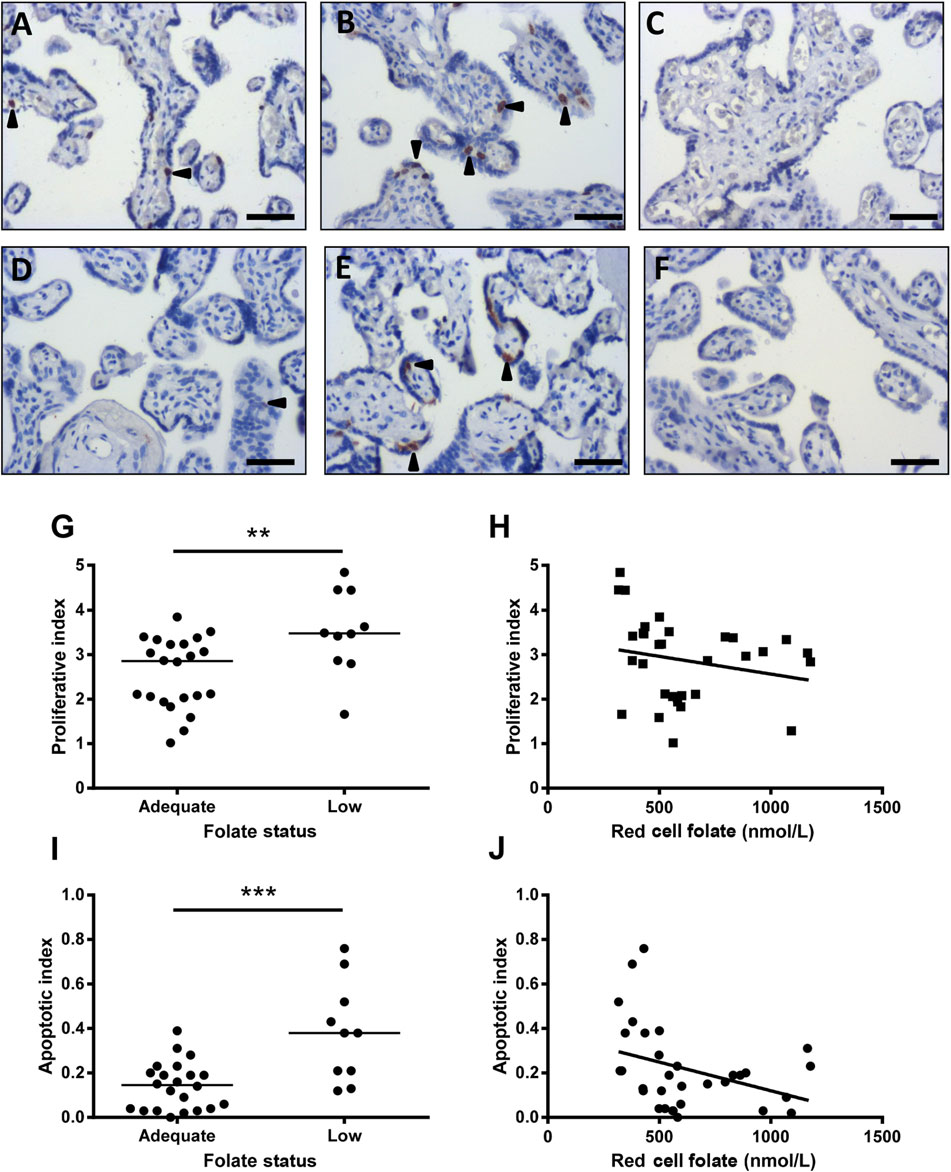
Effect of maternal folate status on placental cell turnover. Representative images of Ki67 and M30 immunohistochemistry in placentas from adolescents with (A and D) adequate (*n* = 24) and (B and E) low (*n* = 10) folate status. (C and F) Negative. Proliferation index (Ki67+ nuclei/total nuclei) was (G) elevated in placentas from women with low versus normal folate status (*p* < 0.01, Mann–Whitney test), and (H) inversely correlated to maternal RBC folate concentrations (*r* = –0.359, *p* < 0.05, Spearman's rank). Apoptotic index (M30+ cells/total nuclei) was (I) elevated in placentas from women with low versus normal folate status (*p* < 0.001, Mann–Whitney test), and (J) inversely related to maternal RBC folate concentrations (*r* = –0.426, *p* ≤ 0.01, Spearman's rank). **p < 0.01, ***p < 0.001. Scale bars = 50 μm. Arrowheads indicate positive staining.

Low maternal folate status was associated with elevated trophoblast proliferation (*p* ≤ 0.01) and apoptosis (*p* ≤ 0.001) compared to placentas from adolescents with adequate folate status (Fig. 1). Both proliferative (*r* = –0.359, *p* < 0.05) and apoptotic (*r* = –0.426, *p* ≤ 0.01) indices negatively correlated with RBC folate.

### 3.4 Impact of maternal folate status on placental nutrient transport

**Figure 2:**
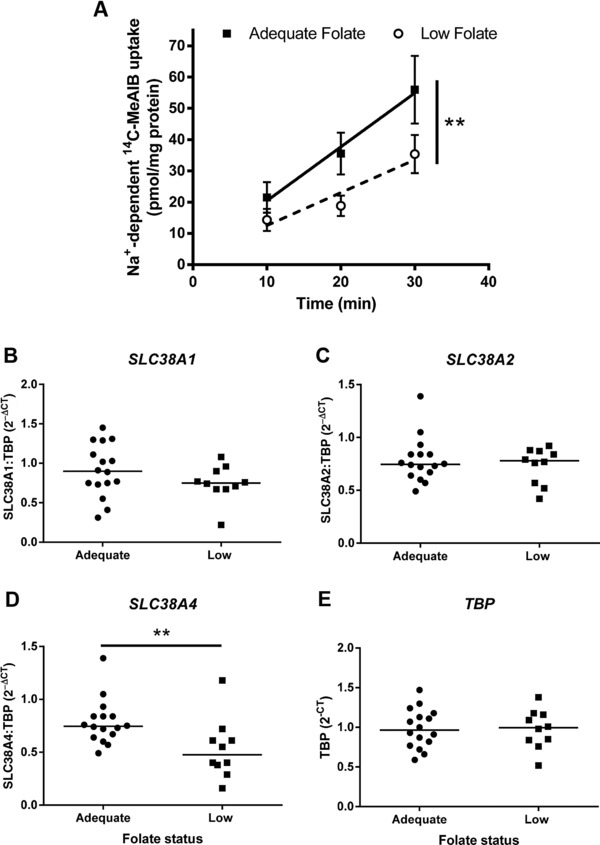
Effect of maternal folate status on placental amino acid transport. (A) System A activity (sodium‐dependent ^14^C‐methylaminoisobutyric acid (^14^C‐MeAIB) uptake) in placental fragments. Mean ± SEM; **p ≤ 0.01, linear regression. Placental mRNA expression of system A isoforms: (B) *SLC38A1*, (C) *SLC38A2*, and (D) *SLC38A4*. Data are 2^–ΔCT^ normalized for (E) TBP (TATA box binding protein). Line represents median, **p < 0.01, Mann–Whitney test. *n* = 12 (adequate), *n* = 10 (low folate status).

System A activity was significantly lower in placentas from adolescents with low compared to adequate folate status (*p* < 0.01; Fig. 2). There was no effect of maternal folate status on nonspecific sodium‐independent ^14^C‐methylaminoisobutyric acid uptake (not shown). Placental mRNA expression of one of the system A isoforms (*SLC38A4*, *p* < 0.01) was lower with low folate status, while SLC38A1 and *2* were unchanged between groups (Fig. 2).

### 3.5 Impact of maternal folate status on placental endocrine function

**Figure 3:**
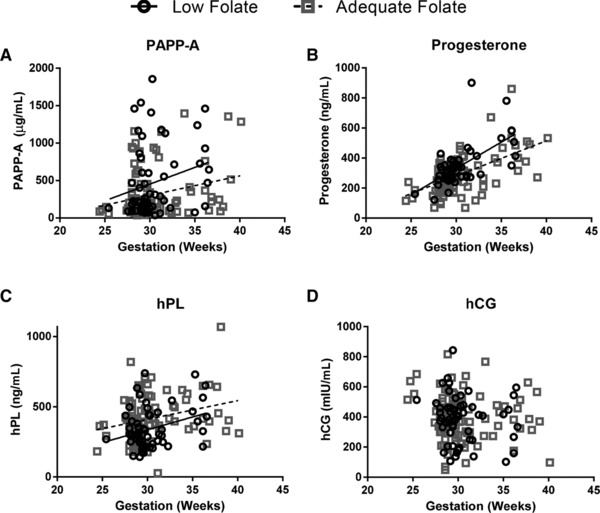
Effect of maternal folate status on placental endocrine function. Positive correlations with gestation detected for (A) pregnancy‐associated plasma protein A (PAPP‐A, *r* ^2^ 0.1973, *p* < 0.05), (B) progesterone (*r* ^2^ 0.5652, *p* < 0.0001), and (C) human placental lactogen (hPL) (*r* ^2^ 0.2008, *p* < 0.01), but not (D) human chorionic gonadotrophin (hCG). Significant differences in the slope (progesterone, *p* < 0.01) or elevation and intercepts (PAPP‐A (*p* < 0.05) and hPL (*p* < 0.01)) were detected between the groups (linear regression). *n* = 100 (adequate), *n* = 57 (low folate status).

Maternal serum concentrations of placental hormones were measured in the third trimester (Fig. 3). PAPP‐A, progesterone, and hPL increased with advancing gestation (*p* < 0.01). Linear regression showed that PAPP‐A and progesterone concentrations were higher in women with low folate status (*p* < 0.05 and *p* < 0.01 respectively), while hPL was lower (*p* < 0.01). hCG concentrations were unchanged across gestation and between folate groups (not shown). Placental mRNA expression of the peptide hormones, PAPP‐A, hPL, hCG (β subunit), and 3β‐hydroxysteroid dehydrogenase (progesterone biosynthetic enzyme) was unaffected by folate status (Supporting Information Fig. 1).

### 3.6 Effect of maternal folate status on expression of known folate‐sensitive miRNAs

**Figure 4:**
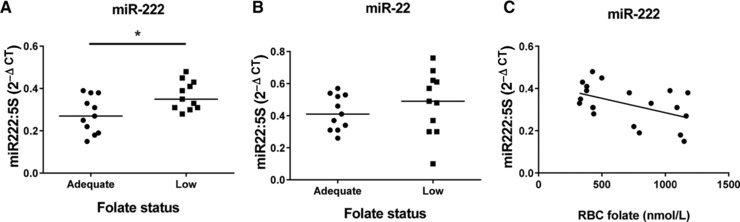
Effect of maternal folate status on placental expression of folate‐sensitive miRNAs. (A) miR‐222‐3p and (B) miR‐22‐5p. *p < 0.05 Mann–Whitney test. (C) Relationship between miR‐222‐3p and RBC folate status (Spearman's correlation, *r* = –0.4908, *p* < 0.05). Data are normalized to 5S and expressed as 2^–ΔCT^. *n* = 11 (adequate), *n* = 11 (low folate status).

miR‐222‐3p expression was higher in placentas from adolescents with low folate status (*p* < 0.05) and negatively correlated with RBC folate (*r* = –0.4908, *p* < 0.05) (Fig. 4A and C). miR‐22‐5p was unaffected by maternal folate status (Fig. 4B), while placental expression of miR‐302a and ‐122 was below the limit of PCR detection (not shown).

### 3.7 Identification of folate‐sensitive miRNAs in placenta

**Figure 5:**
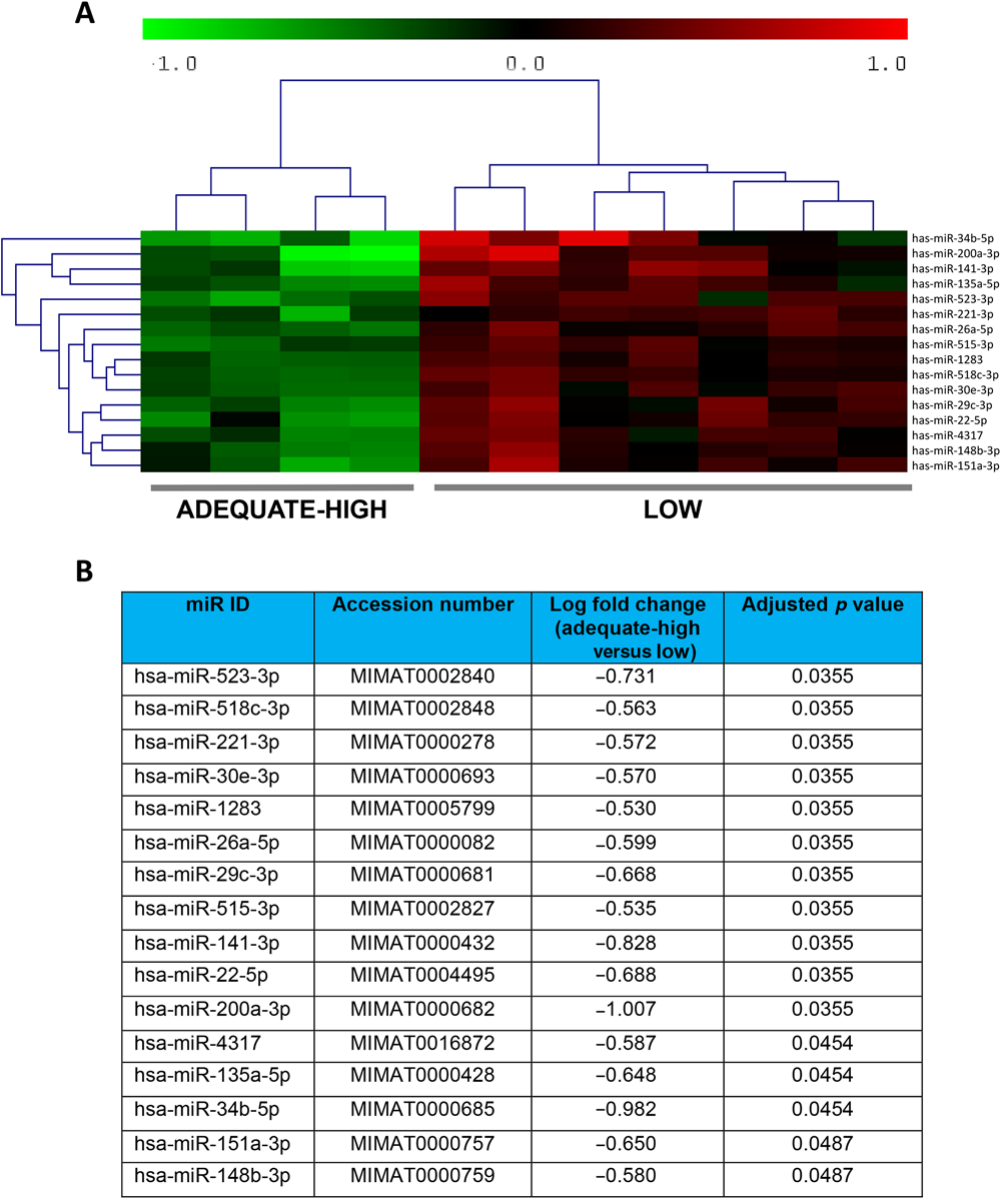
Gene array analysis of miRNAs in placentas according to maternal folate status. (A) Heatmap illustrating 16 differentially expressed miRNAs between women with low (*n* = 7, RBC folate <453 nmol/L) and adequate high folate status (*n* = 4, RBC folate >1000 nmol/L). Each row represents a miRNA and each column a sample. Color scale illustrates relative miRNA expression level, with red below and green above the reference channel. (B) Table showing log fold change (log FC) and *p*‐value for differentially expressed miRNAs (Benjamini and Hochberg multiple test).

**Figure 6:**
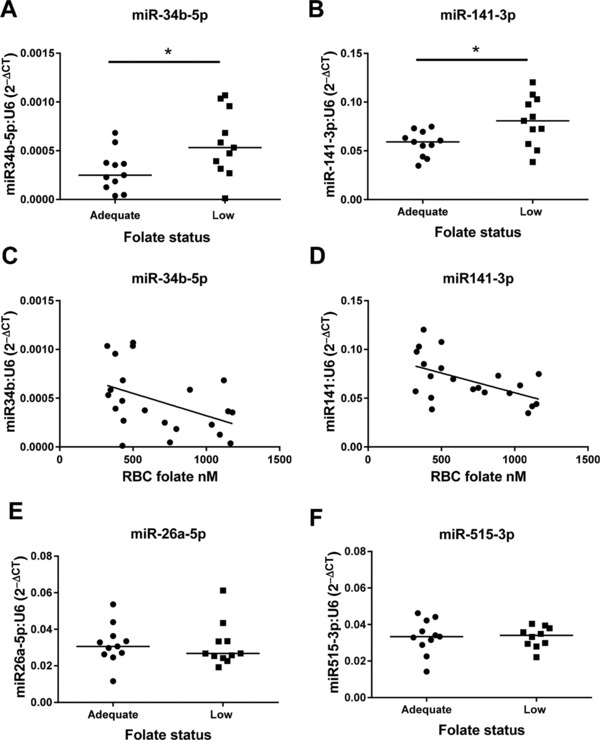
Real‐time PCR validation of differentially expressed placental miRNAs between women with high and low folate status. (A) miR‐34b‐5p, (B) miR‐141‐3p, (E) miR‐26a‐5p, and (F) miR‐515‐3p, *p < 0.05 Mann–Whitney test. Correlation between maternal RBC folate and (C) miR‐34b‐5p (–0.4385, *p* < 0.05) and (D) miR‐141‐3p (–0.4565, *p* < 0.05) Spearman's correlations. Data are normalized to U6 and expressed as 2^–ΔCT,^ *n* = 11/group.

miRNA array expression profiling identified 16 miRNAs that were differentially expressed by maternal folate status. Interestingly, these differences were only apparent between placentas from adolescents with folate status at the higher range of adequate folate status (>1000 nM; “adequate‐high” on Fig. 5) and those with low folate status. The remaining three samples in the adequate group were all from women with folate concentrations between 888 and 965 nM and they had no clear clustering on the heatmap. The 16 miRNAs were all significantly upregulated (*p* < 0.05) by low folate status and included trophoblast‐specific miRNAs: miR‐523‐3p, miR‐518c‐3p, and miR‐515‐3p. miR222 was altered by low folate status, but this narrowly failed to reach statistical significance in this sample set (*p* = 0.06). Six miRNAs (26a‐5p, 29c‐3p, 30e‐3p, 34b‐5p, 141–3p, and 515‐3p) were selected for QPCR validation studies on the basis of high expression by trophoblast cells (determined by performing PCR screens on isolated placental cell types, data not shown) and their previously reported association with pregnancy pathologies 25, 26. While no significant differences were detected in 26a‐5p, 29c‐3p, 30e‐3p, or 515‐3p expression between groups by QPCR, miR‐141‐3p and miR‐34b‐5p were confirmed to be expressed at higher levels in placentas from women with low folate status (*p* < 0.05, Fig. 6). Negative correlations were detected between RBC folate and miR141‐3p (*r* = –0.457, *p* < 0.05) or miR‐34b‐5p (*r* = –0.439, *p* < 0.05) expression (Spearman's correlation; Fig. 6).

### 3.8 Bioinformatic analyses of folate‐sensitive miRNAs

Network generation performed initially on the 16 miRNAs significantly altered from the array revealed biological connectivity between key genes, cyclin‐dependent kinase 6 (CDK6), v‐myc avian myelocytomatosis viral oncogene homolog (MYC), phosphatase and tensin homolog, and insulin (Supporting Information Fig. 2). These genes are known to be important for placental function and fetal growth.

#### 3.8.1 Gene targets of miR‐222‐3p, miR‐34b‐5p, and miR‐141‐3p

**Figure 7:**
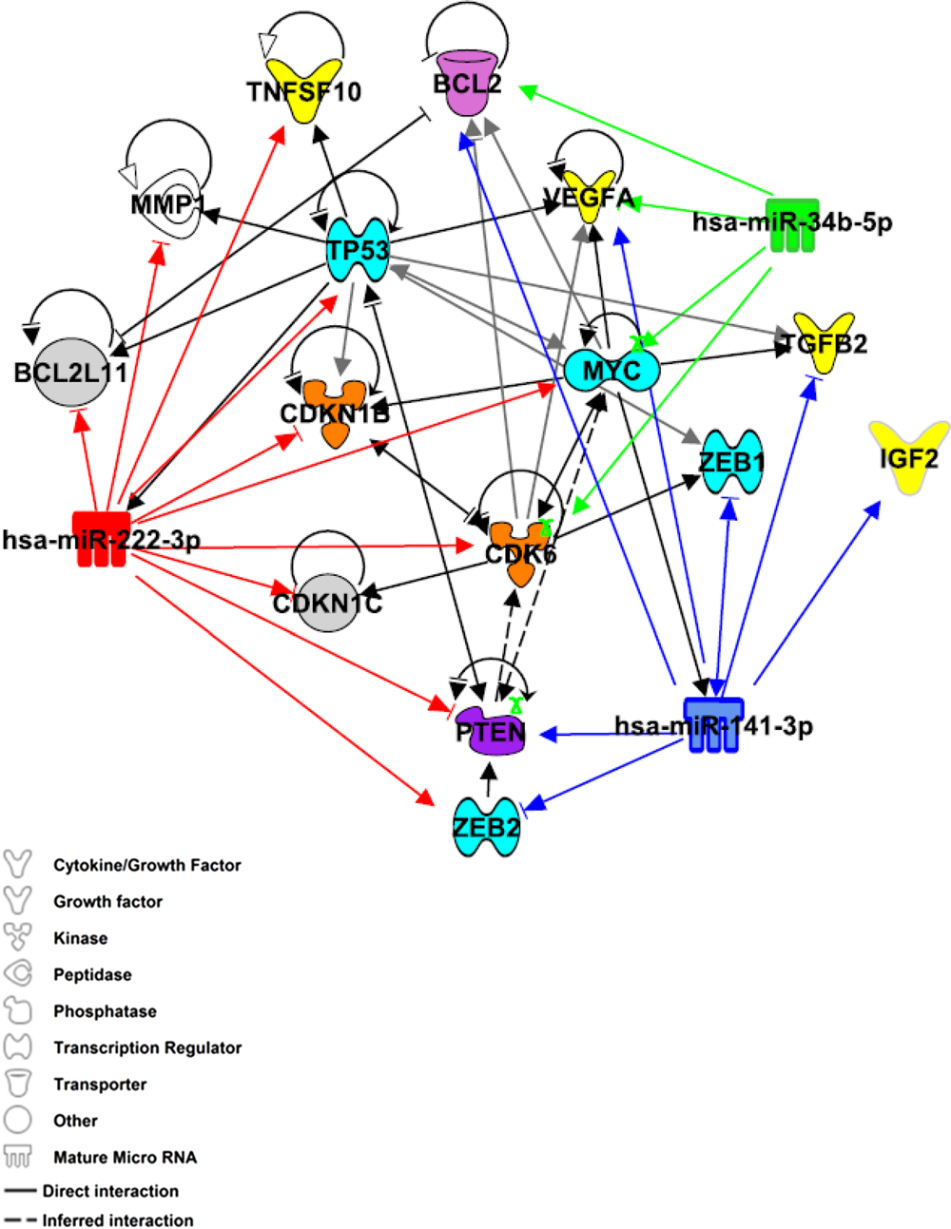
Bioinformatic analyses of miRNAs and their downstream gene targets. A network of known (solid lines) and inferred (dashed lines) interacting molecules was created utilizing the knowledge base within Ingenuity Pathway Analysis. Experimentally validated gene targets of miR‐222‐3p (red), 34b‐5p (green), and 141–3p (blue) retrieved from miRTarBase Release 4.5 23 and TARBASE version 7.0 24 are indicated by solid lines.

Known (experimentally validated) target genes of miR‐222‐3p, 34b‐5p, and 141–3p identified by data mining were refined to 11 genes based on reported association with pregnancy pathologies and/or folate status (Table 3). Genes altered by both folate status and in placentas from FGR pregnancies included MYC 25, 26, vascular endothelial growth factor A (VEGFA) 27, 28, BCL2‐like 11 (BCL2L11/BIM) 28, 29, cyclin‐dependent kinase inhibitor 1C (CDKN1C/p57) 25, 28, and tumor protein 53 (TP53) 28, 30. Other gene targets associated with placental pathologies include homeobox genes zinc finger E‐box binding homeobox 1 (ZEB1) and ZEB2 31 and insulin‐like growth factor 2 32. Many of these genes are targeted by more than one of the candidate miRNAs, therefore the interactions between miRNAs and gene targets were explored by Ingenuity Pathway network analysis. The generated network demonstrated biological connectivity between target genes, in particular TP53, MYC, and CDK6 (Fig. 7). The predicted functional effect of this network is regulation of cell proliferation and survival.

**Table 3:** Gene targets for folate‐sensitive miRNAs

Gene symbol	Gene name	Validated miRNA target(s)	Predicted miRNA target	Altered in pregnancy pathologies	Altered by folate status
MYC	v‐myc myelocytomatosis viral oncogene homolog (avian)	34b‐5p 222–3p		↑mRNA in FGR placenta [25]	↑ in liver of folate‐deficient rat model 26
ZEB 2	zinc finger E‐box binding homeobox 2	141‐3p 222–3p	34b‐5p	↓ uterine tissue mouse model of PTB 31	
CDK6	Cyclin‐dependent kinase 6	34b‐5p	141‐3p 222‐3p	Placental expression positively correlates with birthweight 48	
VEGF A	Vascular endothelial growth factor A	34b‐5p	141‐3p	↑ in FGR placenta 27	↑ or ↓ in folate deficient cells 28
BCL2	B‐cell CLL/lymphoma 2	34b‐5p	141‐3p 222–3p	↑ in FGR/PE placenta 29	
BCL2L11	BCL2‐like 11 (apoptosis facilitator, BIM)	222‐3p	34b‐5p 141‐3p	↑ in FGR placenta 29	↑ with folate deficiency 28
CDKN1B	Cyclin‐dependent kinase inhibitor 1B (p27, Kip1)	222‐3p	34b‐5p 141‐3p	↑ in PE placenta 53	↑ with folate deficiency 28
CDKN1C	Cyclin‐dependent kinase inhibitor 1C (p57, Kip2)	222‐3p		↑ in PE/FGR placenta 25, 53	↑ with folate deficiency 28
TP53	Tumor protein p53	222‐3p	34b‐5p 141‐3p	↑ in FGR placenta 30	↑ and ↓ with folate deficiency 28
ZEB 1	Zinc finger E‐box binding homeobox 1	141‐3p		↓ in uterine tissue mouse model of PTB 31	
IGF2	Insulin like growth factor 2	141‐3p		Placental specific K/O mouse models have FGR pups 32	

Experimentally validated and putative targets of miRNAs, and association with folate deficiency and/or pregnancy complications.

FGR, fetal growth restriction; IBC, individualized birthweight centile; K/O, knock‐out; PE, preeclampsia; PTB, preterm birth.

#### 3.8.2 Expression of predicted targets of folate‐sensitive miRNAs in placentas from women with low folate status

**Figure 8:**
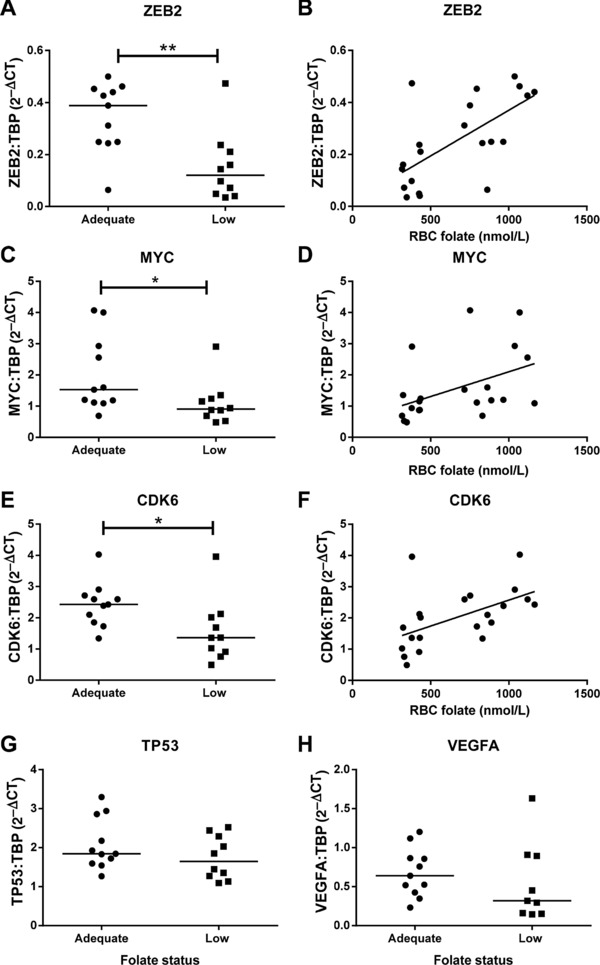
Expression of folate‐sensitive miRNA target genes in placentas of women with low compared to adequate folate status. QPCR analysis of (A) *ZEB2*, (C) *MYC*, (E) *CDK6*, (G) *TP53*, and (H) *VEGFA*. Data are normalized to TBP (TATA box binding protein) and expressed as 2^–ΔCT^. *p < 0.05, **p < 0.01 Mann–Whitney test. Relationship between maternal RBC folate concentration and (B) *ZEB2* (0.6112, *p* < 0.01), (D) *MYC* (0.4961, *p* < 0.05), and (F) *CDK6* (0.6307, *p* < 0.01). Spearman's correlations, *n* = 11 (adequate); *n* = 10 (low folate status).

QPCR analysis of genes (ZEB2 (*p* < 0.01), MYC (*p* < 0.05), and CDK6 (*p* < 0.05)) identified by bioinformatics analysis as known targets of the folate‐sensitive miRNAs demonstrated reduced expression in placentas from adolescents with low maternal folate status (Fig. 8). Positive correlations between maternal RBC folate and ZEB2 (*r* = 0.611, *p* < 0.01), MYC (*r* = 0.496, *p* < 0.05), and CDK6 (*r* = 0.631, *p* < 0.05; Spearman's correlation) mRNA expression were detected. Expression of TP53 and VEGFA were unchanged by maternal folate status.

## 4 Discussion

This study extends our previous observations associating third trimester low maternal folate status with impaired fetal growth 8, supporting the hypothesis that placental dysfunction underlies this process. We demonstrated abnormalities in trophoblast proliferation and apoptosis, nutrient transport, and endocrine function in adolescents with low folate status, and proposed altered miRNA expression as a potential posttranscriptional mechanism.

The placental phenotype in adolescents with low folate status closely resembles pregnancies complicated by FGR/SGA, including elevated trophoblast apoptosis 30 and reduced system A activity 33, 34. Increased apoptosis occurs in trophoblast cultured under folate‐deficient conditions or treated with homocysteine to mimic biochemical consequences of folate deficiency 35, 36. In contrast to FGR 30, we observed elevated proliferation in women with low folate status but in concordance with increased trophoblast proliferation in placentas of compromised fetuses with reduced movements 37 and preeclamptic pregnancies 38. This may reflect placental compensatory responses promoting fetal growth, observed with maternal iron deficiency anemia or smoking 39. Reduced system A activity with low maternal folate status is consistent with observations in both animal models and women with lower maternal nutritional status 20, 34. Placental gene expression of major system A isoforms, *SLC38A1* and *SLC38A2* 40, were unchanged by maternal folate status, suggesting posttranscriptional mechanisms were operating. In contrast, placental SLC38A4 expression was reduced with low folate status, consistent with reports of specific altered mRNA expression in a mouse model of FGR 41 and in women administered synthetic glucocorticoids antenatally 42. The functional consequences of altered SLC38A4 are questionable, however, as the relative contribution of this isoform to overall system A activity in term placentas is very low 40. In common with FGR/SGA, hPL concentrations were significantly lower in low folate status women 43, 44. The elevated concentrations of PAPP‐A and progesterone in women with low folate status imply specific alterations in placental endocrinology, not simply changed placental mass.

Our investigation of posttranscriptional mechanisms for placental dysfunction focused on miRNAs. Although some known folate‐sensitive miRNAs were unaltered in our study, expression of miR‐222‐3p was increased in placentas from low folate status women, consistent with reported upregulation in T lymphocytes in response to folate deficiency 13. Using an unbiased miRNA array, 16 miRNAs, including miR‐34b‐5p and miR‐141‐3p, were altered with low folate status, potentially suggesting these novel folate‐sensitive miRNAs provide a mechanistic posttranscriptional link between maternal folate levels and placental dysfunction. Altered expression of miR‐222‐3p and miR‐141‐3p has been reported in pregnancy pathologies 12, 45, specifically miR‐141‐3p expression is upregulated in FGR placentas. The likely mechanism for elevated miRNA expression under low folate conditions is promoter hypomethylation due to reduced levels of methyl donor S‐adenosylmethionine 46. This is supported by altered methylation and expression of placental miRNAs in a calorie‐restricted mouse model of FGR 47.

Bioinformatics analysis identified predicted gene targets of the folate‐sensitive miRNAs, including genes regulating cell cycle and apoptosis (e.g., TP53, BCL2, MYC, CDKN1B/C, and TNFSF10), consistent with the elevated trophoblast apoptosis observed. Other target genes (e.g., VEGFA, MMP1, insulin‐like growth factor 2) are involved in tissue remodeling, angiogenesis, and placental development, indicating additional placental functions may be affected by maternal folate status. Higher miRNA expression would reduce expression of downstream target genes in placentas from folate‐deficient women; this was confirmed for ZEB2, MYC, and CDK6 at the mRNA level. Placental CDK6 expression positively correlates with birthweight 48, implying reduced expression under low folate conditions would negatively affect fetal growth. Reduced MYC and unaltered TP53 expression in placentas from women with low folate status contrast with FGR pregnancies, where both are elevated 25, 26, 30. This may reflect differences in the placental molecular phenotypes underlying FGR (e.g., hypoxia related to uteroplacental insufficiency versus folate deficiency).

Many putative gene targets are regulated by multiple folate‐sensitive miRNAs, strengthening evidence for their involvement in placental dysfunction and possible additive effects where multiple miRNAs target the same gene. Several target genes have the capacity to regulate diverse downstream networks, as transcription factors, for example, MYC, or phosphatases that modulate cellular signaling (e.g., phosphatase and tensin homolog), potentially amplifying the effect of low folate status on placental function.

A strength of this study is stratification of women based on RBC folate concentration, a more stable marker than serum folate levels, reflecting folate status over a 3‐month period. Study limitations concern the time between blood folate analysis and placental functional measurements at term and variability between subjects, particularly in miRNA studies that are highly susceptible to environmental and fetal parameters (e.g., smoking and fetal gender) 49, 50. This is likely to underlie the discrepancy in the array and PCR findings for miR222, whose upregulation narrowly failed to reach significance on the smaller sample set analyzed by array, and the fact that only two of the six miRNAs identified by array were successfully validated by QPCR. In vitro manipulation of trophoblast folate status would reduce confounding influences providing definitive evidence of direct regulation of miRNAs and target genes by intracellular folate content.

This unique observational study links miRNA expression with folate deprivation in pregnancy highlighting the importance of adequate folate status throughout pregnancy to protect against SGA/FGR. These observations were made using a threshold of 453 nmol/L to define low folate status, and this was validated by our detection of higher rates of SGA births and placental dysfunction below these concentrations. Optimal RBC folate concentrations for fetal growth have not been defined and our observation of clustering of placental miRNA profiles in women with folate status >1000 nM may imply that, at least at a molecular level, adequate folate concentrations for normal placental function greatly exceed those defined for deficiency status in nonpregnant women. This is consistent with recent evidence suggesting that levels >900 nmol/L maximize reduction in occurrence of neural tube defects 17 and protect against uracil misincorporation and apoptosis 51.

In summary, we demonstrate that placental functions critical to optimal fetal growth are altered in adolescents with low folate status in late pregnancy, and this is associated with altered miRNA expression. Whether miRNA mis‐expression also occurs in the fetus is unknown, but it is a potential mechanism for developmental programing as described in animal models of maternal undernutrition 52. Although the current study was conducted on pregnant adolescents, we believe the findings are applicable to the wider obstetric population, reinforcing the need for further mechanistic studies on the impact of maternal nutritional status on the placenta.

*The authors have declared no conflict of interest*.

## Supporting information

Supplementary Figure 1: placental hormone PCR data

Supplementary Figure 2: Network analysis showing miR‐mRNA interactions for the 16 miRs upregulated in low folate status placentas. Direct interactions are denoted by solid lines and predicted by dashed lines. Upregulated miRs are shown in red and 4 key genes known to be important in placental function and fetal growth, CDK6, MYC, PTEN and insulin are highlighted.

**Supplementary Table 1. Primer sequences used for qPCR**. hPL: human placental lactogen, PAPP‐A: Pregnancy associated plasma protein‐A, hCG: human chorionic gonadotropin, HSD: hydroxysteroid dehydrogenase, TBP: TATA‐box binding protein, CDK6: cyclin‐dependent kinase 6, MYC: v‐myc avian myelocytomatosis viral oncogene homolog, ZEB2: zinc finger E‐box binding homeobox 2, TP53: tumor protein p53, VEGFA: vascular endothelial growth factor A.
**Supplementary Table 2: Demographic and biophysical data of TEENs study participants for QPCR analysis**. Median (range) value given unless otherwise stated. BMI: Body Mass Index; IBC: individualised birthweight centile; RBC: red blood cell. Mann‐Whitney test (continuous variables), Fisher's exact test (categorical data).
**Supplementary Table 3. Target sequences used for miRNA specific qPCR**.
**Supplementary Table 4: Demographic and biophysical data of TEENs study participants for miRNA array analysis**. Median (range) value given unless otherwise stated. BMI: Body Mass Index; IBC: individualised birthweight centile; RBC: red blood cell. Mann‐Whitney test (continuous variables), Fisher's exact test (categorical data).
**Supplementary Table 5. Demographic data of TEENs study participants for placental analyses**. Data are median (range) unless otherwise stated. BMI: Body Mass Index at time of booking to antenatal care; RBC: red blood cell. Mann‐Whitney test (continuous data), Fisher's exact test (categorical data). *p<0.05, **p<0.01, ***p<0.0001

## References

[1] Belkacemi, L. , Nelson, D. M. , Desai, M. , Ross, M. G. , Maternal undernutrition influences placental‐fetal development. Biol. Reprod. 2010, 83, 325–331. 20445129 10.1095/biolreprod.110.084517

[2] McIntire, D. D. , Bloom, S. L. , Casey, B. M. , Leveno, K. J. , Birth weight in relation to morbidity and mortality among newborn infants. N Engl J Med 1999, 340, 1234–1238. 10210706 10.1056/NEJM199904223401603

[3] Murray, E. , Fernandes, M. , Fazel, M. , Kennedy, S. H. , et al., Differential effect of intrauterine growth restriction on childhood neurodevelopment: a systematic review. BJOG 2015, 122, 1062–1072. 25990812 10.1111/1471-0528.13435

[4] Barker, D. J. , Winter, P. D. , Osmond, C. , Margetts, B. et al., Weight in infancy and death from ischaemic heart disease. Lancet 1989, 2, 577–580. 2570282 10.1016/s0140-6736(89)90710-1

[5] Schlotz, W. , Phillips, D. I. , Fetal origins of mental health: evidence and mechanisms. Brain Behav Immun 2009, 23, 905–916. 19217937 10.1016/j.bbi.2009.02.001

[6] Berti, C. , Biesalski, H. K. , Gartner, R. , Lapillonne, A. et al., Micronutrients in pregnancy: current knowledge and unresolved questions. Clin. Nutr. 2011, 30, 689–701. 21872372 10.1016/j.clnu.2011.08.004

[7] Stover, P. J. , Field, M. S. , Trafficking of intracellular folates. Adv. Nutr. 2011, 2, 325–331. 22332074 10.3945/an.111.000596 PMC3125682

[8] Baker, P. N. , Wheeler, S. J. , Sanders, T. A. , Thomas, J. E. et al., A prospective study of micronutrient status in adolescent pregnancy. Am. J. Clin. Nutr. 2009, 89, 1114–1124. 19244368 10.3945/ajcn.2008.27097

[9] Fekete, K. , Berti, C. , Trovato, M. , Lohner, S. et al., Effect of folate intake on health outcomes in pregnancy: a systematic review and meta‐analysis on birth weight, placental weight and length of gestation. Nutr. J. 2012, 11, 75. 22992251 10.1186/1475-2891-11-75 PMC3499376

[10] van Uitert, E. M. , Steegers‐Theunissen, R. P. , Influence of maternal folate status on human fetal growth parameters. Mol. Nutr. Food Res. 2013, 57, 582–595. 23213022 10.1002/mnfr.201200084

[11] Sibley, C. P. , Turner, M. A. , Cetin, I. , Ayuk, P. et al., Placental phenotypes of intrauterine growth. Pediatr. Res. 2005, 58, 827–832. 16183820 10.1203/01.PDR.0000181381.82856.23

[12] Fu, G. , Brkic, J. , Hayder, H. , Peng, C. , MicroRNAs in human placental development and pregnancy complications. Int. J. Mol. Sci. 2013, 14, 5519–5544. 23528856 10.3390/ijms14035519 PMC3634453

[13] Marsit, C. J. , Eddy, K. , Kelsey, K. T. , MicroRNA responses to cellular stress. Cancer Res. 2006, 66, 10843–10848. 17108120 10.1158/0008-5472.CAN-06-1894

[14] Liang, Y. , Li, Y. , Li, Z. , Liu, Z. et al., Mechanism of folate deficiency‐induced apoptosis in mouse embryonic stem cells: cell cycle arrest/apoptosis in G1/G0 mediated by microRNA‐302a and tumor suppressor gene Lats2. Int. J. Biochem. Cell Biol. 2012, 44, 1750–1760. 22828209 10.1016/j.biocel.2012.07.014

[15] Mongelli, M. , Gardosi, J. , Reduction of false‐positive diagnosis of fetal growth restriction by application of customized fetal growth standards. Obstet. Gynecol. 1996, 88, 844–848. 8885925 10.1016/0029-7844(96)00285-2

[16] Kim, Y. I. , Fawaz, K. , Knox, T. , Lee, Y. M. et al., Colonic mucosal concentrations of folate are accurately predicted by blood measurements of folate status among individuals ingesting physiologic quantities of folate. Cancer Epidemiol. Biomarkers Prev. 2001, 10, 715–719. 11401925

[17] Cordero, A. M. , Crider, K. S. , Rogers, L. M. , Cannon, M. J. et al., Optimal serum and red blood cell folate concentrations in women of reproductive age for prevention of neural tube defects: World Health Organization guidelines. MMWR Morb Mortal Wkly Rep. 2015, 64, 421–423. 25905896 PMC5779552

[18] Mayhew, T. M. , Taking tissue samples from the placenta: an illustration of principles and strategies. Placenta 2008, 29, 1–14. 17658596 10.1016/j.placenta.2007.05.010

[19] Burton, G. J. , Sebire, N. J. , Myatt, L. , Tannetta, D. et al., Optimising sample collection for placental research. Placenta 2014, 35, 9–22. 24290528 10.1016/j.placenta.2013.11.005

[20] Hayward, C. E. , Greenwood, S. L. , Sibley, C. P. , Baker, P. N. et al., Effect of maternal age and growth on placental nutrient transport: potential mechanisms for teenagers' predisposition to small‐for‐gestational‐age birth? Am J Physiol. Endocrinol Metab 2012, 302, E233–E242. 22028413 10.1152/ajpendo.00192.2011 PMC3340900

[21] Hayward, C. E. , Greenwood, S. L. , Sibley, C. P. , Baker, P. N. et al., Effect of young maternal age and skeletal growth on placental growth and development. Placenta 2011, 32, 990–998. 22005108 10.1016/j.placenta.2011.09.016

[22] Benjamini, Y. , Hochberg, Y. , Controlling the false discovery rate: a practical and powerful approach to multiple testing. J. R. Stat. Soc. Ser. B 1995, 57, 289–300.

[23] Hsu, S. D. , Tseng, Y. T. , Shrestha, S. , Lin, Y. L. et al., miRTarBase update 2014: an information resource for experimentally validated miRNA‐target interactions. Nucleic Acids Res. 2014, 42, D78–D85. 24304892 10.1093/nar/gkt1266 PMC3965058

[24] Vlachos, I. S. , Paraskevopoulou, M. D. , Karagkouni, D. , Georgakilas, G. et al., DIANA‐TarBase v7.0: indexing more than half a million experimentally supported miRNA:mRNA interactions. Nucleic Acids Res. 2015, 43, D153–D159. 25416803 10.1093/nar/gku1215 PMC4383989

[25] Rajaraman, G. , Murthi, P. , Pathirage, N. , Brennecke, S. P. et al., Downstream targets of homeobox gene HLX show altered expression in human idiopathic fetal growth restriction. Am. J. Pathol. 2010, 176, 278–287. 20008130 10.2353/ajpath.2010.090187 PMC2797890

[26] Dizik, M. , Christman, J. K. , Wainfan, E. , Alterations in expression and methylation of specific genes in livers of rats fed a cancer promoting methyl‐deficient diet. Carcinogenesis 1991, 12, 1307–1312. 2070497 10.1093/carcin/12.7.1307

[27] Roh, C. R. , Budhraja, V. , Kim, H. S. , Nelson, D. M. et al., Microarray‐based identification of differentially expressed genes in hypoxic term human trophoblasts and in placental villi of pregnancies with growth restricted fetuses. Placenta 2005, 26, 319–328. 15823618 10.1016/j.placenta.2004.06.013

[28] Novakovic, P. , Stempak, J. M. , Sohn, K. J. , Kim, Y. I. , Effects of folate deficiency on gene expression in the apoptosis and cancer pathways in colon cancer cells. Carcinogenesis 2006, 27, 916–924. 16361273 10.1093/carcin/bgi312

[29] Whitehead, C. L. , Walker, S. P. , Lappas, M. , Tong, S. , Circulating RNA coding genes regulating apoptosis in maternal blood in severe early onset fetal growth restriction and pre‐eclampsia. J. Perinatol. 2013, 33, 600–604. 23429544 10.1038/jp.2013.16

[30] Heazell, A. E. P. , Sharp, A. N. , Baker, P. N. , Crocker, I. P. , Intra‐uterine growth restriction is associated with increased apoptosis and altered expression of proteins in the p53 pathway in villous trophoblast. Apoptosis 2011, 16, 135–144. 21052841 10.1007/s10495-010-0551-3

[31] Renthal, N. E. , Chen, C. C. , Williams, K. C. , Gerard, R. D. et al., miR‐200 family and targets, ZEB1 and ZEB2, modulate uterine quiescence and contractility during pregnancy and labor. Proc. Natl. Acad. Sci. USA 2010, 107, 20828–20833. 21079000 10.1073/pnas.1008301107 PMC2996411

[32] Constancia, M. , Hemberger, M. , Hughes, J. , Dean, W. et al., Placental‐specific IGF‐II is a major modulator of placental and fetal growth. Nature 2002, 417, 945–948. 12087403 10.1038/nature00819

[33] Glazier, J. D. , Cetin, I. , Perugino, G. , Ronzoni, S. et al., Association between the activity of the system A amino acid transporter in the microvillous plasma membrane of the human placenta and severity of fetal compromise in intrauterine growth restriction. Pediatr. Res. 1997, 42, 514–519. 9380446 10.1203/00006450-199710000-00016

[34] Jansson, N. , Pettersson, J. , Haafiz, A. , Ericsson, A. et al., Down‐regulation of placental transport of amino acids precedes the development of intrauterine growth restriction in rats fed a low protein diet. J. Physiol. 2006, 576, 935–946. 16916910 10.1113/jphysiol.2006.116509 PMC1892642

[35] Di Simone, N. , Riccardi, P. , Maggiano, N. , Piacentani, A. et al., Effect of folic acid on homocysteine‐induced trophoblast apoptosis. Mol. Hum. Reprod. 2004, 10, 665–669. 15286211 10.1093/molehr/gah091

[36] Steegers‐Theunissen, R. P. , Smith, S. C. , Steegers, E. A. , Guilbert, L. J. et al., Folate affects apoptosis in human trophoblastic cells. BJOG. 2000, 107, 1513–1515. 11192109 10.1111/j.1471-0528.2000.tb11677.x

[37] Warrander, L. K. , Batra, G. , Bernatavicius, G. , Greenwood, S. L. et al., Maternal perception of reduced fetal movements is associated with altered placental structure and function. PLoS One 2012, 7, e34851. 22523561 10.1371/journal.pone.0034851 PMC3327709

[38] Arnholdt, H. , Meisel, F. , Fandrey, K. , Lohrs, U. , Proliferation of villous trophoblast of the human placenta in normal and abnormal pregnancies. Virchows Arch. B Cell Pathol Incl Mol Pathol 1991, 60, 365–372. 1683053 10.1007/BF02899568

[39] McNamara, H. , Hutcheon, J. A. , Platt, R. W. , Benjamin, A. et al., Risk factors for high and low placental weight. Paediatr. Perinat. Epidemiol. 2014, 28, 97–105. 24354883 10.1111/ppe.12104

[40] Desforges, M. , Mynett, K. J. , Jones, R. L. , Greenwood, S. L. et al., The SNAT4 isoform of the system A amino acid transporter is functional in human placental microvillous plasma membrane. J. Physiol. 2009, 587, 61–72. 19015196 10.1113/jphysiol.2008.161331 PMC2667314

[41] Constancia, M. , Angiolini, E. , Sandovici, I. , Smith, P. et al., Adaptation of nutrient supply to fetal demand in the mouse involves interaction between the Igf2 gene and placental transporter systems. Proc. Natl. Acad. Sci. USA 2005, 102, 19219–19224. 16365304 10.1073/pnas.0504468103 PMC1316882

[42] Audette, M. C. , Challis, J. R. , Jones, R. L. , Sibley, C. P. et al., Synthetic glucocorticoid reduces human placental system A transport in women treated with antenatal therapy. J. Clin. Endocrinol. Metab. 2014, 99, E2226–E2233. 25105735 10.1210/jc.2014-2157

[43] Dutton, P. J. , Warrander, L. K. , Roberts, S. A. , Bernatavicius, G. et al., Predictors of poor perinatal outcome following maternal perception of reduced fetal movements—a prospective cohort study. PLoS One 2012, 7, e39784. 22808059 10.1371/journal.pone.0039784 PMC3394759

[44] Chowen, J. A. , Evain‐Brion, D. , Pozo, J. , Alsat, E. et al., Decreased expression of placental growth hormone in intrauterine growth retardation. Pediatr Res 1996, 39, 736–739. 8848353 10.1203/00006450-199604000-00028

[45] Tang, Q. , Wu, W. , Xu, X. , Huang, L. et al., miR‐141 contributes to fetal growth restriction by regulating PLAG1 expression. PLoS One 2013, 8, e58737. 23554918 10.1371/journal.pone.0058737 PMC3598866

[46] Stover, P. J. , Polymorphisms in 1‐carbon metabolism, epigenetics and folate‐related pathologies. J Nutrigenet. Nutrigenomics 2011, 4, 293–305. 22353665 10.1159/000334586 PMC3696357

[47] Chen, P. Y. , Ganguly, A. , Rubbi, L. , Orozco, L. D. et al., Intrauterine calorie restriction affects placental DNA methylation and gene expression. Physiol Genomics 2013, 45, 565–576. 23695884 10.1152/physiolgenomics.00034.2013 PMC3727019

[48] Sedlmeier, E. M. , Brunner, S. , Much, D. , Pagel, P. et al., Human placental transcriptome shows sexually dimorphic gene expression and responsiveness to maternal dietary n‐3 long‐chain polyunsaturated fatty acid intervention during pregnancy. BMC Genomics 2014, 15, 941. 25348288 10.1186/1471-2164-15-941 PMC4232618

[49] Maccani, M. A. , Avissar‐Whiting, M. , Banister, C. E. , McGonnigal, B. et al., Maternal cigarette smoking during pregnancy is associated with downregulation of miR‐16, miR‐21, and miR‐146a in the placenta. Epigenetics 2010, 5, 583–589. 20647767 10.4161/epi.5.7.12762 PMC2974801

[50] Muralimanoharan, S. , Guo, C. , Myatt, L. , Maloyan, A. , Sexual dimorphism in miR‐210 expression and mitochondrial dysfunction in the placenta with maternal obesity. Int. J. Obes. 2015, 39, 1274–1281. 10.1038/ijo.2015.45 PMC4526386 25833255

[51] Fenech, M. , Folate (vitamin B9) and vitamin B12 and their function in the maintenance of nuclear and mitochondrial genome integrity. Mutat. Res. 2012, 733, 21–33. 22093367 10.1016/j.mrfmmm.2011.11.003

[52] Fernandez‐Twinn, D. S. , Constancia, M. , Ozanne, S. E. , Intergenerational epigenetic inheritance in models of developmental programming of adult disease. Semin. Cell Dev. Biol. 2015, 43, 85–95. 26135290 10.1016/j.semcdb.2015.06.006 PMC5844462

[53] Unek, G. , Ozmen, A. , Mendilcioglu, I. , Simsek, M. et al., The expression of cell cycle related proteins PCNA, Ki67, p27 and p57 in normal and preeclamptic human placentas. Tissue Cell 2014, 46, 198–205. 24852133 10.1016/j.tice.2014.04.003

