# Supplementary material for: Placental dysfunction is associated with altered microRNA expression in pregnant women with low folate status
Source: Mol Nutr Food Res. 2017 Mar 21;61(8):1600646. doi: 10.1002/mnfr.201600646 (PMC5573923; doi:10.1002/mnfr.201600646)
Supplement: Supplementary file 1 — Supplementary Figure 1: placental hormone PCR data [file MNFR-61-na-s001.pptx]

## Slide 1
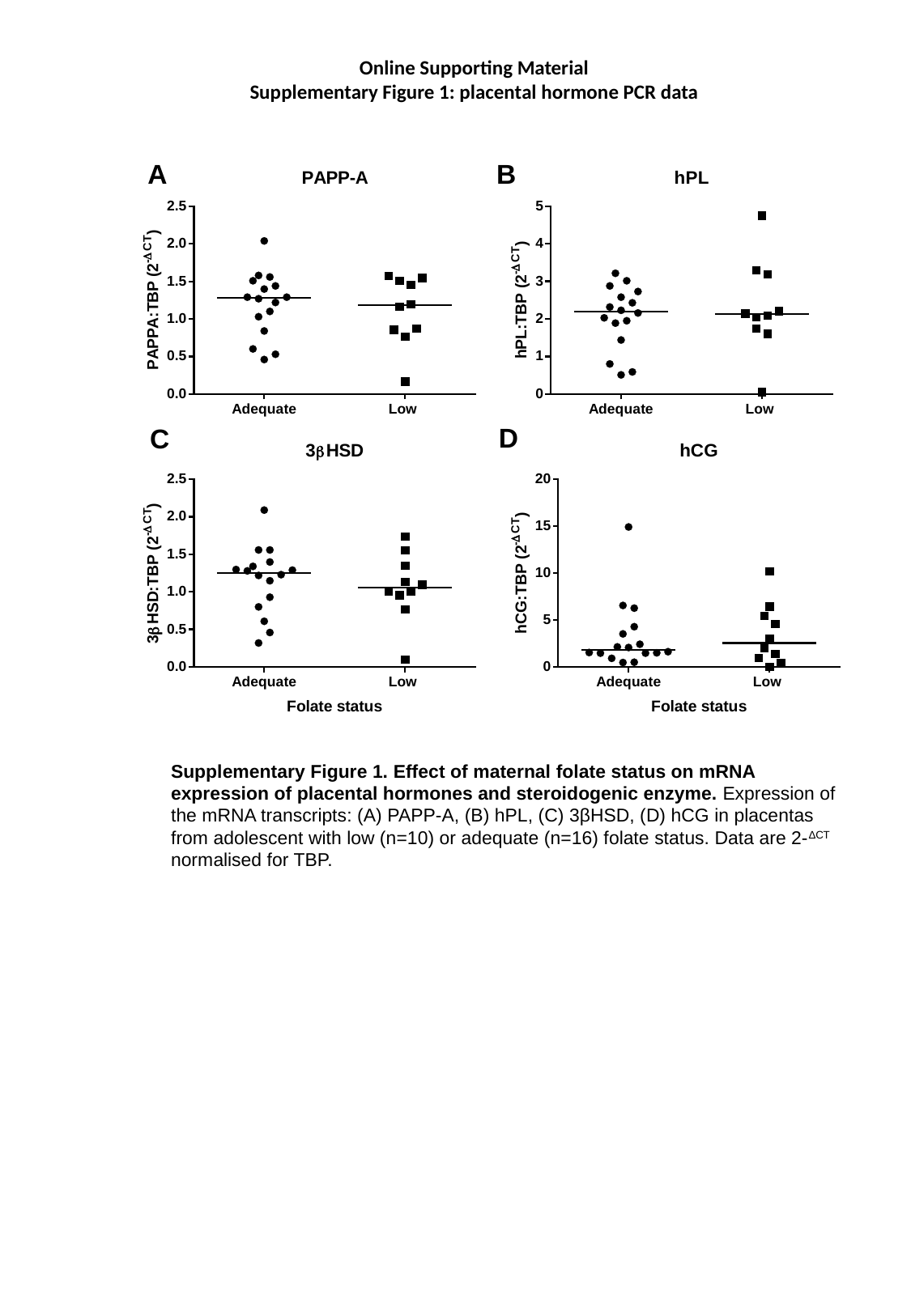

Online Supporting Material
Supplementary Figure 1: placental hormone PCR data
Supplementary Figure 1. Effect of maternal folate status on mRNA expression of placental hormones and steroidogenic enzyme. Expression of the mRNA transcripts: (A) PAPP-A, (B) hPL, (C) 3βHSD, (D) hCG in placentas from adolescent with low (n=10) or adequate (n=16) folate status. Data are 2-ΔCT normalised for TBP.
