# Supplementary material for: Placental dysfunction is associated with altered microRNA expression in pregnant women with low folate status
Source: Mol Nutr Food Res. 2017 Mar 21;61(8):1600646. doi: 10.1002/mnfr.201600646 (PMC5573923; doi:10.1002/mnfr.201600646)
Supplement: Supplementary file 2 — Supplementary Figure 2: Network analysis showing miR‐mRNA interactions for the 16 miRs upregulated in low folate status placentas. Direct interactions are denoted by solid lines and predicted by dashed lines. Upregulated miRs are shown in red and 4 key genes known to be important in placental function and fetal growth, CDK6, MYC, PTEN and insulin are highlighted. [file MNFR-61-na-s002.pptx]

## Slide 1
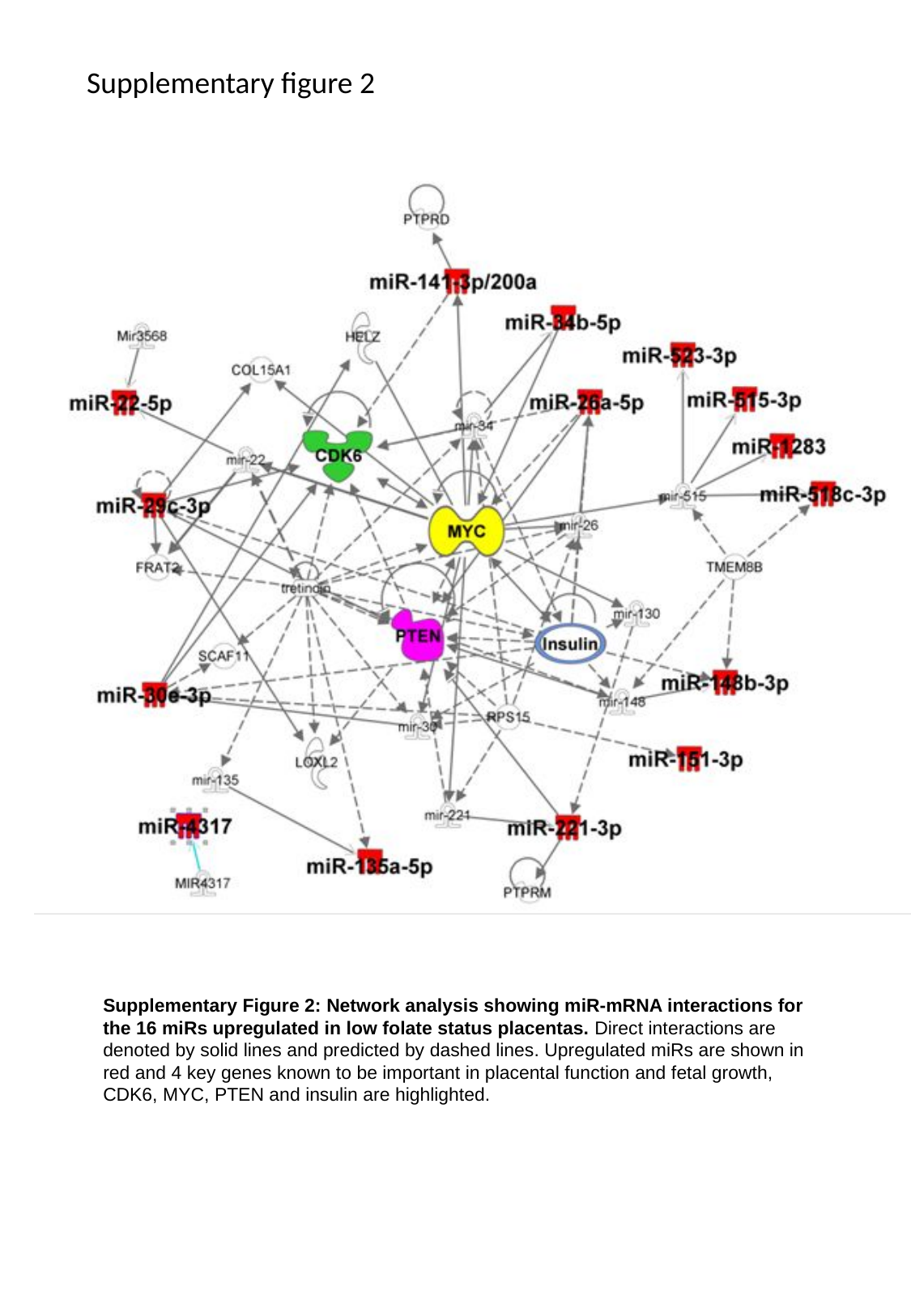

Supplementary figure 2
Supplementary Figure 2: Network analysis showing miR-mRNA interactions for the 16 miRs upregulated in low folate status placentas. Direct interactions are denoted by solid lines and predicted by dashed lines. Upregulated miRs are shown in red and 4 key genes known to be important in placental function and fetal growth, CDK6, MYC, PTEN and insulin are highlighted.
