# Supplementary material for: Placental dysfunction is associated with altered microRNA expression in pregnant women with low folate status
Source: Mol Nutr Food Res. 2017 Mar 21;61(8):1600646. doi: 10.1002/mnfr.201600646 (PMC5573923; doi:10.1002/mnfr.201600646)
Supplement: Supplementary file 3 — Supplementary Table 1. Primer sequences used for qPCR. hPL: human placental lactogen, PAPP‐A: Pregnancy associated plasma protein‐A, hCG: human chorionic gonadotropin, HSD: hydroxysteroid dehydrogenase, TBP: TATA‐box binding protein, CDK6: cyclin‐dependent kinase 6, MYC: v‐myc avian myelocytomatosis viral oncogene homolog, ZEB2: zinc finger E‐box binding homeobox 2, TP53: tumor protein p53, VEGFA: vascular endothelial growth factor A. Supplementary Table 2: Demographic and biophysical data of TEENs study participants for QPCR analysis. Median (range) value given unless otherwise stated. BMI: Body Mass Index; IBC: individualised birthweight centile; RBC: red blood cell. Mann‐Whitney test (continuous variables), Fisher's exact test (categorical data). Supplementary Table 3. Target sequences used for miRNA specific qPCR. Supplementary Table 4: Demographic and biophysical data of TEENs study participants for miRNA array analysis. Median (range) value given unless otherwise stated. BMI: Body Mass Index; IBC: individualised birthweight centile; RBC: red blood cell. Mann‐Whitney test (continuous variables), Fisher's exact test (categorical data). Supplementary Table 5. Demographic data of TEENs study participants for placental analyses. Data are median (range) unless otherwise stated. BMI: Body Mass Index at time of booking to antenatal care; RBC: red blood cell. Mann‐Whitney test (continuous data), Fisher's exact test (categorical data). *p<0.05, **p<0.01, ***p<0.0001 [file MNFR-61-na-s003.docx]

| **Gene** | **Primer Sequence (5’ – 3’)** | **Accession No.** |
| --- | --- | --- |
| **SLC38A1** | F: GTGTATGCTTTACCCACCATTGC  R: GCACGTTGTCATAGAATGTCAAGT | NM_030674 |
| **SLC38A2** | F: ACGAAACAATAAACACCACCTTAA  R: AGATCAGAATTGGCACAGCATA | NM_018976 |
| **SLC38A4** | F: TTGCCGCCCTCTTTGGTTAC  R: GAGGACAATGGGCACAGTTAGT | NM_018018 |
| **hPL** | F: TCCTCAGGAGTATGT  R: CACAGCTACCCTCTA | NM_020991 |
| **PAPP-A** | F: TGAAGCTCTATGTGAATGGTGC  R: TGTAGCCCCGGTAGTTGTGAT | NM_002581 |
| **β-hCG subunit** | F: TCACTTCACCGTGGTCTCCG  R: TGCAGCACGCGGGTCATGGT | NM_000737 |
| **3β-HSD** | F: TAACGGGTGGAATCTGAAAAACG  R: CTAGCAGAAAGGAATCGGCTTC | NM_000862 |
| **TBP** | F: CACGAACCACGGCACTGATT  R: TTTTCTTGCTGCCAGTCTGGAC | NM_001172085 |
| **CDK6** | F: TCTTCATTCACACCGAGTAGTGC  R: TGAGGTTAGAGCCATCTGGAAA | NM_001145306 |
| **MYC** | F: GTCAAGAGGCGAACACACAAC  R: TTGGACGGACAGGATGTATGC | NM_002467 |
| **ZEB2** | F: TCTGTAGATGGTCCAGTGAAGA  R: GTCACTGCGCTGAAGGTACT | NM_001171653.1 |
| **TP53** | F: CAGCACATGACGGAGGTTGT  R: TCATCCAAATACTCCACACGC | NM_001126118 |
| **VEGFA** | F: CGCAGCTACTGCCATCCAAT  R: GTGAGGTTTGATCCGCATAATCT | NM_001025366.2 |

**Supplementary Table 1. Primer sequences used for qPCR.**hPL: human placental lactogen, PAPP-A: Pregnancy associated plasma protein-A, hCG: human chorionic gonadotropin, HSD: hydroxysteroid dehydrogenase, TBP: TATA-box binding protein, CDK6: cyclin-dependent kinase 6, MYC: v-myc avian myelocytomatosis viral oncogene homolog, ZEB2: zinc finger E-box binding homeobox 2, TP53: tumor protein p53, VEGFA: vascular endothelial growth factor A.

| **Category** | **Adequate Folate status (n=11)** | **Low Folate status**  **(n=11)** | ***p*** |
| --- | --- | --- | --- |
| **Age**  years | 18.2 (15.6-18.9) | 17.7 (15.9-18.6) | *NS* |
| **Gynaecological age**  years | 5.2  (2.8-6.9) | 4.7  (2.2-7.6) | *NS* |
| **Caucasian**  n (%) | 10 (91%) | 8 (73%) | *NS* |
| **Primiparous**  n (%) | 11  (100%) | 10  (91%) | *NS* |
| **Smoker**  n (%) | 3 (27%) | 5 (45%) | *NS* |
| **BMI at booking**  kg/m^2^ | 24.6 (18.8 – 28.3) | 21.0 (17.1 – 24.3) | *p<0.05* |
| **Gestation**  weeks | 40.4 (36.3-41.1) | 41 (39.4-41.7) | *NS* |
| **Birthweight**  g | 3430  (2350 - 3790) | 3375  (2920 - 4160) | *NS* |
| **IBC** | 40(6 – 99) | 35 (8 – 98) | *NS* |
| **Time from blood sampling to delivery**  weeks | 7.72 (2.72 – 12.85) | 7.72 (2.29 -12.57) | *NS* |
| **Interval between eating and blood sampling**  (hrs) | 2.17 (0.5 – 19.45) | 3.2 (1.3 – 17.3) | *NS* |
| **Male Infant**  n (%) | 3 (43%) | 4 (57%) | *NS* |
| **Serum folate**  nmol/L | 12.7 (6.3 – 29) | 6.1 (4.5 – 11.8) | *p<0.01* |
| **RBC folate**  nmol/L | 888 (580 – 1178) | 426 (324 - 498) | *p<0.0001* |

**Supplementary Table 2: Demographic and biophysical data of TEENs study participants for QPCR analysis.** Median (range) value given unless otherwise stated. BMI: Body Mass Index; IBC: individualised birthweight centile; RBC: red blood cell. Mann-Whitney test (continuous variables), Fisher’s exact test (categorical data).

| **miRNA Primer set** | **Target Sequence (5’ – 3’)** | **Accession no.** |
| --- | --- | --- |
| **hsa-miR-515-3p** | GAGUGCCUUCUUUUGGAGCGUU | MIMAT0002827 |
| **hsa-miR-30e-3p** | CUUUCAGUCGGAUGUUUACAGC | MIMAT0000693 |
| **hsa-miR-34b-5p** | UAGGCAGUGUCAUUAGCUGAUUG | MIMAT0000685 |
| **hsa-miR-222-3p** | AGCUACAUCUGGCUACUGGGU | MIMAT0000279 |
| **hsa-miR-29c-3p** | UAGCACCAUUUGAAAUCGGUUA | MIMAT0000681 |
| **hsa-miR-26a-5p** | UUCAAGUAAUCCAGGAUAGGCU | MIMAT0000082 |
| **hsa-miR-22-5p** | AGUUCUUCAGUGGCAAGCUUUA | MIMAT0004495 |
| **hsa-miR-141-3p** | UAACACUGUCUGGUAAAGAUGG | MIMAT0000432 |
| **hsa-miR-143-3p** | UGAGAUGAAGCACUGUAGCUC | MIMAT0000435 |
| **hsa-miR-145-5p** | GUCCAGUUUUCCCAGGAAUCCCU | MIMAT0000437 |
| **hsa-miR-302a-3p** | UAAGUGCUUCCAUGUUUUGGUGA | MIMAT0000684 |
| **hsa-miR-122-5p** | UGGAGUGUGACAAUGGUGUUUG | MIMAT0000421 |

**Supplementary Table 3. Target sequences used for miRNA specific qPCR.**

| **Category** | **Adequate Folate status (n=7)** | **Low Folate status**  **(n=7)** | ***p*** |
| --- | --- | --- | --- |
| **Age**  *years* | 18 (15-18) | 17 (15-18) | *NS* |
| **Gynaecological age**  years | 5.3  (3.5-6.9) | 4.3  (2.2-6.6) | *NS* |
| **Caucasian**  *n* (%) | 6 (86%) | 5 (71%) | *NS* |
| **Primiparous**  n (%) | 7  (100%) | 7  (100%) | *NS* |
| **Smoker**  *n* **(%)** | 1 (14%) | 2 (28%) | *NS* |
| **BMI at booking**  kg/m^2^ | 24.97 (17.5 – 28.2) | 21.2 (17.1 – 24.3) | *p<0.05* |
| **Gestation**  weeks | 39.1 (37.4-40.8) | 41 (39.4-41.7) | *NS* |
| **Birthweight**  g | 3460  (2350 - 3790) | 3375  (2500 - 4160) | *NS* |
| **IBC** | 44 (16 – 99) | 35 (4 – 98) | *NS* |
| **Male Infant**  *n* **(%)** | 3 (43%) | 4 (57%) | *NS* |
| **Serum folate**  nmol/L | 14.3 (7.3 – 29) | 6.1 (3.4 – 11.1) | *p<0.01* |
| **RBC folate**  nmol/L | **1038** (831 – 1119) | **346** (317-428) | *p<0.001* |

**Supplementary Table 4: Demographic and biophysical data of TEENs study participants for miRNA array analysis.** Median (range) value given unless otherwise stated. BMI: Body Mass Index; IBC: individualised birthweight centile; RBC: red blood cell. Mann-Whitney test (continuous variables), Fisher’s exact test (categorical data).

| **Category** | **Adequate Folate status** (n=22) | **Low Folate status**  (n=10) | ***p*** |
| --- | --- | --- | --- |
| **Age**  *Years* | 18.1 (15.6-18.9) | 17.7 (15.9-18.6) | *NS* |
| **Gynaecological age**  *Years* | 5.2 (2.2-8.7) | 4.9 (2.2-7.6) | *NS* |
| **Ethnicity***n (%)*  Caucasian  Other | 18 (81.8%)  4 (18.2%) | 8 (80%)  2 (20%) | *NS* |
| **Primiparous**  *n (%)* | 21 (95.5%) | 10 (100%) | *NS* |
| **Smoking status** *n (%)*  Smoker  Non-smoker | 9 (40.9%)  13 (59.1%) | 5 (50%)  5 (50%) | *NS*  *NS* |
| **BMI**  *kg/m^2^* | 24.6 (18.7-28.3) | 20.8 (17.1-24.3) | *<0.05* |
| **Gestation at delivery**  weeks | 40.5 (36.3-42.0) | 40.8 (39.4-41.7) | *NS* |
| **Birthweight**  g | 3445 (2020-4320) | 3370 (2760-4160) | *NS* |
| **Individualised Birthweight Centile** (IBC) | 40.5 (0-99) | 32 (4-98) | *NS* |
| **Male Infant**  *n (%)* | 8 (36.4%) | 4 (40%) | *NS* |
| **Serum folate**  *nmol/L* | 9.2 (5.7-29.0) | 6.0 (3.4-11.1) | < 0.01 |
| **RBC folate**  *nmol/L* | 631.1 (498.5-1178.3) | 379.6 (317.2-435.1) | < 0.0001 |

**Supplementary Table 5. Demographic data of TEENs study participants for placental analyses.** Data are median (range) unless otherwise stated. BMI: Body Mass Index – at time of booking to antenatal care; RBC: red blood cell. Mann-Whitney test (continuous data), Fisher’s exact test (categorical data). *p<0.05, **p<0.01, ***p<0.0001
